# Successful Use of Midodrine in a Normotensive, Non-cirrhotic Patient With Chylous Ascites Due to Chronic Porto-Mesenteric Thrombosis: A Multidisciplinary Challenge

**DOI:** 10.7759/cureus.96307

**Published:** 2025-11-07

**Authors:** Samer Alhasan, Hisham Tharwat, Nazima Salim, Ramia Alhasan, Nadiah S Alsafar, Azeez Alzafiri

**Affiliations:** 1 Internal Medicine, Al Jahra Hospital, Al Jahra, KWT

**Keywords:** chylous ascites, midodrine, normotensive, portal vein thrombosis, superior mesenteric vein thrombosis

## Abstract

Chylous ascites (CA), the pathological accumulation of triglyceride-rich lymphatic fluid in the peritoneal cavity, is rare and most often associated with cirrhosis, malignancy, trauma, or lymphatic injury. Its occurrence in non-cirrhotic patients with chronic portal- and superior mesenteric vein (SMV) thrombosis is exceedingly uncommon, and therapeutic options remain limited.

We describe a 43-year-old Kuwaiti woman with a history of sleeve gastrectomy, complicated by portal and SMV thrombosis, and later laparoscopic cholecystectomy. She presented with progressive abdominal distension and pain. Imaging revealed chronic porto-mesenteric thrombosis, and paracentesis confirmed CA with a triglyceride level of 2.56 mmol/L. Comprehensive evaluation excluded malignancy, cirrhosis, tuberculosis, and lymphatic leakage. Initial conservative therapy, including a low-fat, high-protein diet supplemented with medium-chain triglycerides, total parenteral nutrition, and subcutaneous octreotide, was only partially effective, and octreotide was discontinued due to elevated liver enzymes. Because of persistent ascites, oral midodrine was initiated at 7.5 mg twice daily and later titrated to 7.5 mg three times daily, despite normotension. The patient showed marked improvement, with complete resolution at one-month follow-up. This case highlights the pathophysiological link between chronic porto-mesenteric venous obstruction, splanchnic hypertension, and CA. Midodrine, through α1-adrenergic agonism, likely reduced splanchnic lymphatic pressure and chyle leakage. Although conventionally reserved for hypotension, midodrine demonstrated therapeutic benefit in a normotensive patient. It may represent a novel adjunct in the management of refractory CA due to portal vein thrombosis, underscoring the importance of a multidisciplinary approach and the need for further research into vasoconstrictor therapy in this setting.

## Introduction

Chylous ascites (CA) is an uncommon form of ascites, defined by the accumulation of triglyceride-rich lymphatic fluid, known as chyle, within the peritoneal cavity [1]. Chyle is normally composed of lymph and dietary fats absorbed from the intestinal lacteals and transported through the thoracic duct into the systemic circulation [2]. When this transport pathway is disrupted or overwhelmed, lymphatic fluid accumulates in the peritoneal space, giving rise to the characteristic milky appearance of CA and leading to abdominal distension, discomfort, and nutritional compromise [3]. The causes of CA are diverse and may be congenital or acquired. In adults, the most common etiologies include malignancy, particularly lymphoma and pancreatic cancer, and cirrhosis with portal hypertension, which increases lymphatic fluid production and impairs drainage [4,5]. Other important causes are abdominal trauma and surgery, where damage to lymphatic vessels may result in chyle leakage [5], as well as infections, such as abdominal tuberculosis [5]. Inflammatory conditions, like pancreatitis, and rare congenital lymphatic malformations are also recognized contributors. The diagnosis of CA is established by biochemical analysis of ascitic fluid, typically when the triglyceride concentration exceeds accepted thresholds. The diagnosis is confirmed by ascitic fluid triglyceride levels above 1.1 mmol/L [6], although more recent papers define the threshold for diagnosis as a triglyceride content greater than 200 mg/dL [7]. Management generally follows a stepwise approach, beginning with dietary modification - such as a low-fat, high-protein diet with medium-chain triglyceride (MCT) supplementation - and pharmacological therapy, including somatostatin analogues, in addition to addressing the underlying cause [8].

The patient described in this report presented a diagnostic challenge because the usual etiologies - malignancy, tuberculosis, lymphatic leakage, and cirrhosis - were systematically excluded. The eventual diagnosis implicated chronic portal and superior mesenteric vein (SMV) thrombosis, a rare vascular cause of CA. This case highlights the importance of conservative management, the potential therapeutic role of midodrine, even in normotensive patients, and the value of a multidisciplinary approach in guiding successful outcomes.

## Case presentation

A 43-year-old Kuwaiti woman with no past medical history presented with progressive abdominal distention and recurrent upper abdominal pain for one month. Her surgical history included sleeve gastrectomy in 2018, complicated by portal and SMV thrombosis managed conservatively, and laparoscopic cholecystectomy in 2022, again complicated by thrombosis. In 2022, she was prescribed direct oral anticoagulants for six months but discontinued them after one month because of missed hematology follow-up appointments. There was no history of trauma, oral contraceptive use, or chronic medication use. She was admitted to the surgical ward on May 9, 2024, following multiple emergency department visits for abdominal distention and pain. On examination, she was alert, oriented, afebrile, and hemodynamically stable. Abdominal examination revealed generalized tenderness and guarding. A contrast-enhanced computed tomography (CT) of the abdomen revealed chronic portal and SMV thrombosis with collateral formation, progressive ascites, splenomegaly, and incidental right colonic diverticulitis.

**Figure 1:**
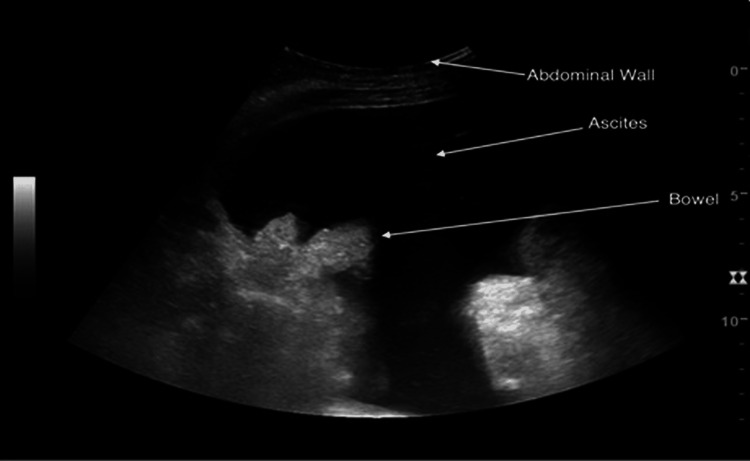
An ultrasound image showing moderate ascites Abdominal ultrasound, performed with a curvilinear probe (LOGIQ E9 system; GE HealthCare, Wauwatosa, WI, USA), demonstrates a large anechoic (dark) fluid collection within the peritoneal cavity, consistent with ascites. The abdominal wall is visualized superficially at the top, while echogenic bowel loops are seen floating within the ascitic fluid. This image highlights the characteristic sonographic appearance of ascites.

On admission, the patient underwent a thorough clinical and radiological assessment. Physical examination revealed a distended abdomen with shifting dullness, but no features of hypotension. Abdominal ultrasound confirmed the presence of moderate ascites, as demonstrated in Figure 1. Diagnostic paracentesis was performed under ultrasound guidance, yielding 2.5 L of opaque, milky fluid. Biochemical analysis of the ascitic fluid revealed a triglyceride level of 2.56 mmol/L (greater than 200 mg/dL), which confirmed the diagnosis of CA. The serum-ascites albumin gradient was elevated, supporting the presence of portal hypertension. Cytological examination of the fluid showed no malignant cells.

A systematic evaluation was undertaken to exclude common causes of CA. Tuberculosis was ruled out by negative polymerase chain reaction (PCR), negative mycobacterial cultures, and a negative QuantiFERON test. Viral and other infectious etiologies, including cytomegalovirus and hepatitis viruses, were excluded by serological testing. Autoimmune screening was negative, and there was no laboratory evidence of connective tissue disease. Malignancy was excluded by negative ascitic cytology, cross-sectional contrast-enhanced imaging, and fluorodeoxyglucose positron emission tomography-computed tomography (FDG PET-CT), all of which revealed no suspicious lesions. Cirrhosis was considered unlikely, as blood counts and coagulation profiles were normal, liver biochemistry was within reference limits, and imaging showed no morphological stigmata of chronic liver disease.

Finally, a lymphangiogram performed with Lipiodol injection into the inguinal lymphatics showed no extravasation into the peritoneum, excluding an active lymphatic leak. Instead, Doppler ultrasound and contrast-enhanced CT imaging confirmed the presence of chronic portal and SMV thrombosis with collateral formation, which was identified as the underlying vascular pathology. An upper gastrointestinal endoscopy revealed features of portal hypertensive gastropathy and Grade II esophageal varices, which were subsequently ligated.

The patient’s case was reviewed in a multidisciplinary team (MDT) meeting that included specialists in surgery, gastroenterology, hematology, vascular medicine, nutrition, and internal medicine. In the absence of sepsis, peritonitis, or evidence of surgically correctable lymphatic disruption, conservative management was recommended. Because of the dual risks of gastrointestinal bleeding from varices and the absence of acute thrombotic progression, anticoagulation was withheld following risk-benefit discussion within the MDT.

Conservative therapy was initiated to reduce chyle formation and support nutritional status. A high-protein, low-fat diet enriched with MCT oil was prescribed at a dose of 10 mL orally twice daily. This dietary modification aimed to reduce long-chain fat absorption via lymphatics, thereby lowering chyle production, as MCTs are directly absorbed through the portal circulation. Given ongoing protein loss and the risk of malnutrition, total parenteral nutrition was also started under specialist dietary supervision. Pharmacological therapy with subcutaneous octreotide, 100 micrograms three times daily, was administered to decrease splanchnic blood flow and inhibit gastrointestinal lymphatic secretions. Despite these measures, octreotide had to be discontinued on the fourth day of treatment because of a significant rise in liver transaminases, with alanine aminotransferase (ALT) increasing from 54 U/L to 89 U/L.

Following the withdrawal of octreotide, the patient continued to accumulate chylous fluid. At this stage, the MDT considered alternative therapeutic options. Given the chronic porto-mesenteric venous thrombosis and its hemodynamic effects, the use of midodrine was discussed as a potential off-label intervention. Although conventionally reserved for hypotensive states, midodrine was introduced at a dose of 7.5 mg orally twice daily and titrated to 7.5 mg three times daily. The rationale was based on its α1-adrenergic agonist properties, which could induce splanchnic vasoconstriction, reduce lymphatic engorgement, and thereby mitigate chyle leakage. The patient tolerated the medication well, with no episodes of hypertension, bradycardia, or other adverse effects.

Following the initiation of midodrine, the patient’s condition improved progressively. Abdominal girth stabilized, oral intake was maintained, and no further paracentesis was required during the hospitalization. She was discharged home in stable condition, and outpatient follow-up one month later revealed complete resolution of ascites on physical examination and abdominal ultrasound, with no clinical recurrence.

## Discussion

Portal vein thrombosis (PVT) represents the blockage or narrowing of the portal vein due to thrombus formation. The portal vein is the major vessel responsible for transporting blood from the gastrointestinal tract, gallbladder, pancreas, and spleen to the liver. Obstruction of this vessel disrupts hepatic blood flow and can give rise to several complications, including portal hypertension, ascites, and, in rare instances, CA [9,10].

The etiologies of PVT are multifactorial and include chronic liver disease, prothrombotic conditions, intra-abdominal infections, and prior abdominal surgery. Surgical procedures can alter vascular anatomy or predispose patients to thrombotic events, as seen in this case. In the context of CA, chronic PVT acts as a direct driver of elevated splanchnic venous and lymphatic pressures, leading to abnormal lymphatic drainage and chyle leakage into the peritoneal cavity.

The standard therapeutic approach to PVT generally consists of anticoagulation to limit thrombus extension and encourage recanalization, typically with agents such as low-molecular-weight heparin or vitamin K antagonists, for a defined course of therapy. In this patient, however, the decision to withhold anticoagulation was made following multidisciplinary discussion, balancing the risks of gastrointestinal bleeding against the absence of acute thrombotic progression. This decision reflects the importance of individualized treatment strategies in patients with both thrombosis and portal hypertensive complications [11].

This case also provides unique insights into the pathophysiology of CA associated with chronic porto-mesenteric venous obstruction. Unlike the more typical associations of CA with cirrhosis or lymphatic disruption, this patient’s normotensive status and absence of chronic liver disease indicated a different underlying mechanism. The longstanding thrombotic occlusion resulted in increased resistance within the splanchnic vasculature, which secondarily elevated intestinal lymphatic pressure. This, in turn, promoted chyle leakage into the peritoneal cavity and contributed to persistent ascites [12].

An additional key feature of this case was the therapeutic use of midodrine. Traditionally employed for conditions such as orthostatic hypotension or hepatorenal syndrome, midodrine exerts its effect through an α1-adrenergic agonist, producing systemic and regional vasoconstriction. In this patient, despite normotension, midodrine was introduced as an adjunctive therapy after failure of dietary measures and octreotide. Its beneficial effect can be explained by its action on the splanchnic circulation, reducing transcapillary filtration, lowering lymphatic pressure, and thereby decreasing chyle leakage. This mechanism may also enhance mesenteric lymphatic drainage and improve vascular tone, even without systemic hypotension. The patient’s favorable response underscores a potentially novel role for midodrine in the management of refractory CA due to porto-mesenteric venous obstruction [13,14].

**Figure 2:**
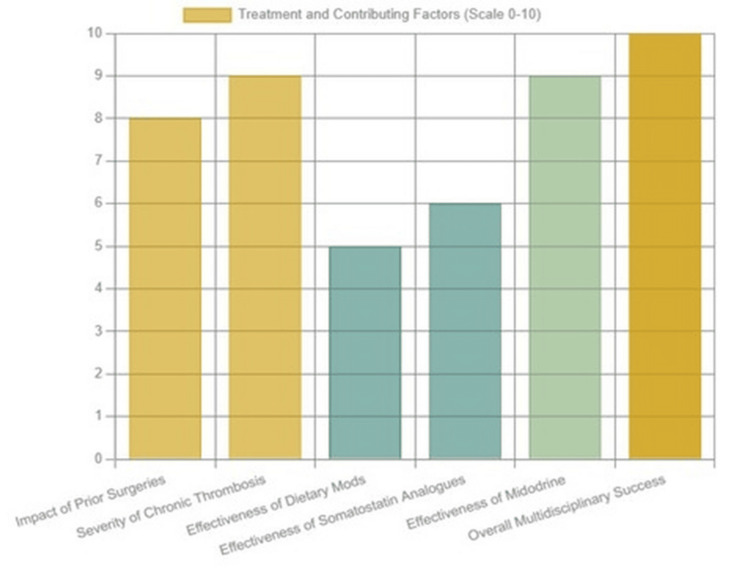
Bar chart illustrating the perceived impact of various factors in this patient’s case on a scale from 0 to 10 It shows the significant roles of prior surgeries and chronic thrombosis as contributing factors, the moderate effectiveness of dietary modifications and somatostatin analogues on their own, the high effectiveness of midodrine, and the ultimate success achieved through an integrated, multidisciplinary approach.

The most distinctive feature of this case was the successful management achieved through a multidisciplinary and non-invasive strategy, supported by structured medical therapy, as illustrated in Figure 2. Octreotide was initially employed because of its established ability to reduce lymphatic flow, but it had to be discontinued when the patient developed elevated liver enzymes. This created a therapeutic gap that necessitated consideration of alternative approaches. Midodrine was introduced despite the absence of systemic hypotension, and its use proved to be pivotal in the patient’s recovery. By enhancing splanchnic vasoconstriction, midodrine likely reduced the formation of lymph and improved lymphatic drainage, thereby alleviating the accumulation of chyle in the peritoneal cavity. Alongside pharmacologic therapy, dietary adjustments played a central role. The prescription of a high-protein, low-fat diet enriched with MCTs reduced chyle production and helped to maintain the patient’s nutritional status during her illness. The patient’s positive response, characterized by stabilization of abdominal girth, absence of paracentesis requirement, and resolution of ascites at follow-up, underscores the potential role of midodrine in selected normotensive individuals. While the evidence base remains limited, this experience suggests that midodrine may serve as a novel adjunctive therapy in the management of CA associated with portal hypertension when standard conservative measures are insufficient.

## Conclusions

This case report highlights the diagnostic and therapeutic challenges of managing CA in a normotensive patient with chronic portal and SMV thrombosis. The successful use of midodrine as an adjunct therapy not only expands the current clinical understanding of treatment options but also suggests that therapies traditionally used for other conditions may have potential benefits in complex and atypical presentations. This case emphasizes that venous outflow obstruction can lead to CA even in the absence of hypotension, challenging conventional thinking. Importantly, it underscores the critical role of a multidisciplinary approach - bringing together specialists from hepatology, surgery, nutrition, and interventional radiology - to ensure accurate diagnosis and comprehensive management. Further research is needed to better understand the mechanisms by which midodrine benefits such patients and to identify those who are most likely to respond to this therapeutic strategy.
